# VASN knockout induces myocardial fibrosis in mice by downregulating non-collagen fibers and promoting inflammation

**DOI:** 10.3389/fphar.2024.1500617

**Published:** 2025-01-17

**Authors:** Junming Sun, Siwei Yin, Qiurui Li, Jun Zhang, Xiaoping Guo, Na Yu, Bing Hu, Yiqiang Ouyang, Qiaojuan Huang, Min He

**Affiliations:** ^1^ Laboratory Animal Center, Guangxi Medical University, Nanning, Guangxi, China; ^2^ Department of Cardiology, The Second Affiliated Hospital, Guangxi Medical University, Nanning, Guangxi, China; ^3^ School of Public Health, Guangxi Medical University, Nanning, China; ^4^ Ministry of Education, Key Laboratory of High-Incidence-Tumor Prevention and Treatment, Guangxi Medical University, Nanning, China

**Keywords:** myocardial fibrosis, vasorin, non-collagen fibers, inflammation, mice

## Abstract

Myocardial fibrosis (MF) is an important cause of heart failure and cardiac arrest. Vasorin knockout (VASN^−/−^) leads to pathological cardiac hypertrophy (PCH); however, it is not yet clear whether this PCH transitions to MF in mice. VASN-knockout mice showed typical pathological, imaging, and molecular features of MF upon hematoxylin and eosin staining, Masson staining, Sirius red staining, quantitative polymerase chain reaction (qPCR), immunohistochemistry-paraffin (IHC-P), and immunofluorescence analyses. RNA was extracted from mouse heart tissue, identified, and sequenced *in vitro*. Differential analysis of the genes showed that the extracellular matrix (ECM) genes (*COL6A1*, *COL9A1,* and *FRAS1*) had strong correlations while their expression levels were significantly reduced by qPCR, IHC-P, and Western blotting. The expression levels of the ECM genes were significantly reduced but those of the inflammatory factors (IL1β and IL6) were significantly upregulated in the heart tissues of VASN-knockout mice. These preliminary results reveal that VASN knockout induces MF by regulating the non-collagen fibers and inflammation.

## 1 Introduction

Myocardial fibrosis (MF) is a key stage of heart failure that can exacerbate the associated symptoms and lead to severe outcomes, such as cardiac arrest or sudden death (López et al., 2021). MF is typically caused by prolonged pressure on or damage to the myocardial cells and often causes cardiac hypertrophy. MF is a complex pathological process that is closely related to the extracellular matrix (ECM), immune responses, signaling pathways, and various cardiac cells (Li et al., 2022). Damaged myocardial cells can activate local inflammatory reactions and release pro-inflammatory cytokines, such as interleukin (IL) 1, IL6, IL11, IL17, and tumor necrosis factor alpha (TNFα). Fibroblasts are activated and transformed into myofibroblasts in the heart tissues, which then synthesize and secrete large amounts of collagen and ECM components (Liu et al., 2021). Collectively, these risk factors contribute to MF.

Vasorin (VASN), also known as slit-like 2 (slitl2), contains two exons, of which exon 2 is the main coding region (Pintus et al., 2018). VASN is a transmembrane glycoprotein composed of 673 amino acids and is located on the cell surface (Bonnet et al., 2018). VASN is highly expressed in the cardiovascular system, including the heart, vascular smooth muscles, and umbilical vein endothelial cells (Bonnet et al., 2018; Pintus et al., 2018). Upregulation of VASN expression prevents smooth muscle cell calcification through specific binding to the transforming growth factor (Luong et al., 2019). Downregulation of VASN expression can alleviate adverse reactions to vascular wall injury (Li et al., 2015). However, overexpression or knockout of VASN has been found to cause developmental abnormalities in the heart and blood vessels of zebrafish (Chen et al., 2005). VASN-knockout (VASN^−/−^) mice have been reported to die suddenly 3 weeks after birth (Ikeda et al., 2004). Our previous study showed that a VASN-knockout mouse model exhibited pathological cardiac hypertrophy symptoms (Sun et al., 2022).

In the present study, VASN-knockout mice showed the pathological, molecular, and protein features of MF. RNA from the mouse heart tissue was extracted, identified, and sequenced *in vitro*. Bioinformatic analysis then showed significantly decreased expressions of key ECM genes (*COL6A1*, *COL9A1*, and *FRAS1*); however, the expressions of inflammatory factors IL1β and IL6 were significantly upregulated in the heart tissues of VASN-knockout mice. Our results thus reveal that VASN knockout induces MF by affecting the ECM and inflammation.

## 2 Materials and methods

### 2.1 Preparation and identification of VASN-knockout mice

All mouse experiments were approved by the Ethics Committee of Guangxi Medical University (approval no. 202209200). C57BL/6J mice were obtained from the Laboratory Animal Center of Guangxi Medical University (SCXK GUI 2020–0003, SYXK GUI 2020–0004). When the VASN^−/−^ mice were 28 days old and exhibited behavioral and morphological characteristics, such as arched backs, sparse hair, reduced body sizes, and immobility, the VASN^+/+^, VASN^+/−,^, and VASN^−/−^ mice from the same batch were divided into three groups for subsequent experiments. The hydroxyproline (HYP) assay was then performed according to manufacturer instructions (A030-2-1; Nanjing Jiancheng) (Sun et al., 2022).

### 2.2 Hematoxylin and eosin (HE) staining

HE staining was performed on the tissue samples from the mice according to a previously described protocol (Sun et al., 2022).

### 2.3 Masson staining

The heart samples were fixed in Bouin’s solution and embedded in paraffin. The slices were then dewaxed, oxidized with 1% potassium permanganate for 5 min, bleached with oxalic acid for 1 min, stained with azure blue for 5 min, dried with Mayer’s hematoxylin for 3–5 min, rinsed under running water for 5–10 min, stained with Lichun red picric acid saturated solution for 5 min, differentiated using 1% phosphomolybdic acid for approximately 5 min, dried with 1% light green for 30 s, differentiated using 95% alcohol, dehydrated with anhydrous ethanol, made transparent with xylene, and lastly sealed with neutral gum.

### 2.4 Sirius staining

The wax layers were first removed from the paraffin sections. Then, iron hematoxylin staining solution was applied to each section for 5–10 min followed by washing with distilled water for 10–20 s. The samples were then soaked in tap water for 5–10 min and cleaned with distilled water thrice for 5–10 s each time. Sirius red staining solution was then applied for 15–30 min, and each section was rinsed gently with running water to remove the surface dye. The slices were rapidly dehydrated using 80%, 95%, and anhydrous ethanol. Finally, the slices were sequentially made transparent in three cylinders of xylene for 3 min before being sealed with neutral gum.

### 2.5 Transcriptome sequencing and bioinformatics analysis

Transcriptome sequencing of the hearts from the three groups was performed at the Wuhan Genome Institute (BGI-Shenzhen), where a total of 12 RNA samples (three mice per group) were sequenced. Data from the whole transcriptome were collected and compared with the ribosome database to identify known transcripts (mRNA), perform quantitative analysis of the known and new mRNAs, and analyze differences between the samples (at least two samples) and groups (at least two samples with at least three biological repeats in each group). The differentially expressed genes (DEGs) were analyzed using the DAVID database through gene ontology (GO) and Kyoto encyclopedia of genes and genomes (KEGG) functional enrichment analyses based on the miRNA target genes.

### 2.6 Quantitative polymerase chain reaction (qPCR) analysis

RNA reverse transcription and qPCR were performed according to a previous study (Sun et al., 2022) with primers (Table 1) obtained from Sangon Biotech (Shanghai, China). Each mRNA was subjected to 40 cycles of PCR, and this process was repeated thrice. The expression levels of the endogenous *GAPDH* genes were compared, and the relative mRNA expressions were compared using the 2^–△△CT^ method.

**TABLE 1:** List of primer sequences.

Gene	Forward/reverse	Sequence
*COL1A1*	Forward	CTG​ACT​GGA​AGA​GCG​GAG​AG
	Reverse	ACA​TTA​GGC​GCA​GGA​AGG​TC
*COL3A1*	Forward	AGC​CTT​CTA​CAC​CTG​CTC​CT
	Reverse	CGG​ATA​GCC​ACC​CAT​TCC​TC
*CTGF*	Forward	AGA​ACT​GTG​TAC​GGA​GCG​TG
	Reverse	GTG​CAC​CAT​CTT​TGG​CAG​TG
*COL6A1*	Forward	ATG​TGC​TCC​TGC​TGT​GAG​TG
	Reverse	TCT​TGC​ATC​TGG​TTG​TGG​CT
*COL9A1*	Forward	CGA​CCG​ACC​AGC​ACA​TCA​A
	Reverse	AGG​GGG​ACC​CTT​AAT​GCC​T
*FRAS1*	Forward	GCT​TGT​CTG​TAT​CAG​GGC​TCC
	Reverse	CTT​CTC​CCT​TCT​CAA​AGG​CAC
*COL2A1*	Forward	AAG​GGA​GAG​ACT​GGA​CCT​GC
	Reverse	GAA​TCC​ACG​GTT​GCC​AGG​AG
*IL1β*	Forward	TGC​AGC​TGG​AGA​GTG​TGG​A
	Reverse	GGC​TTG​TGC​TCT​GCT​TGT​GA
*IL6*	Forward	CTG​CAA​GAG​ACT​TCC​ATC​CAG
	Reverse	AGT​GGT​ATA​GAC​AGG​TCT​GTT​GG
*TNF*	Forward	GAC​GTG​GAA​CTG​GCA​GAA​GAG
	Reverse	TTG​GTG​GTT​TGT​GAG​TGT​GAG
*IL10*	Forward	ACT​ATG​CCG​TCA​GCG​ATA​CAG
	Reverse	GGC​ACC​AGC​TTT​GAA​TAA​TAC​GA
*GAPDH*	Forward	AGG​TCG​GTG​TGA​ACG​GAT​TTG
	Reverse	AGG​AGC​GAG​ACC​CCA​CTA​ACA

### 2.7 Western blotting (WB) analysis

WB was performed according to a previous protocol (Sun et al., 2022) using the primary antibodies COL6A1 (17023-1-AP, Protein, 1:300), COL9A1 (12507-1-AP, Protein, 1:300), FRAS1 (29654-1-AP, Protein, 1:300), COL2A1 (A19308, ABclonal, 1:300), IL1β (D220820, Sangon Biotech, 1:300), IL6 (26404-1-AP, Protein, 1:300), IL10 (60269-1-Ig, Protein, 1:300), TNFα (17590-1-AP, Protein, 1:300), endogenous protein tubulin (AC001, ABclonal, 1:500), and secondary antibodies (AS014, ABclonal, 1:1000). The expressions of the target proteins were calculated using an automatic analysis system (Image Lab 6.0).

### 2.8 Immunohistochemistry (IHC) and immunofluorescence (IF) analyses

IHC-paraffin (IHC-P) and IF analyses were performed according to previously described protocols (Sun et al., 2022; Sun et al., 2021) using the primary antibodies against COL1A1 (A22090, ABclonal, 1:300), COL3A1 (22734-1-AP, Protein, 1:300), and CTGF (25474-1-AP, Protein, 1:300) as well as secondary antibodies (AS014, ABclonal, 1:500). Primary antibodies against α-SMA (67735-1-Ig, protein, 1:200) and a horseradish peroxidase (HRP)-conjugated secondary antibody (AS014, ABclonal, 1:500) were also used.

### 2.9 Statistical analysis

All experiments were performed in triplicate. The data were presented as mean ± standard deviation (SD) and analyzed statistically using one-way analysis of variance (ANOVA) in SPSS software. The value *p* < 0.05 was considered to indicate a significant difference, and *p* < 0.01 indicated an extremely significant difference.

## 3 Results

### 3.1 VASN knockout induces MF

**FIGURE 1:**
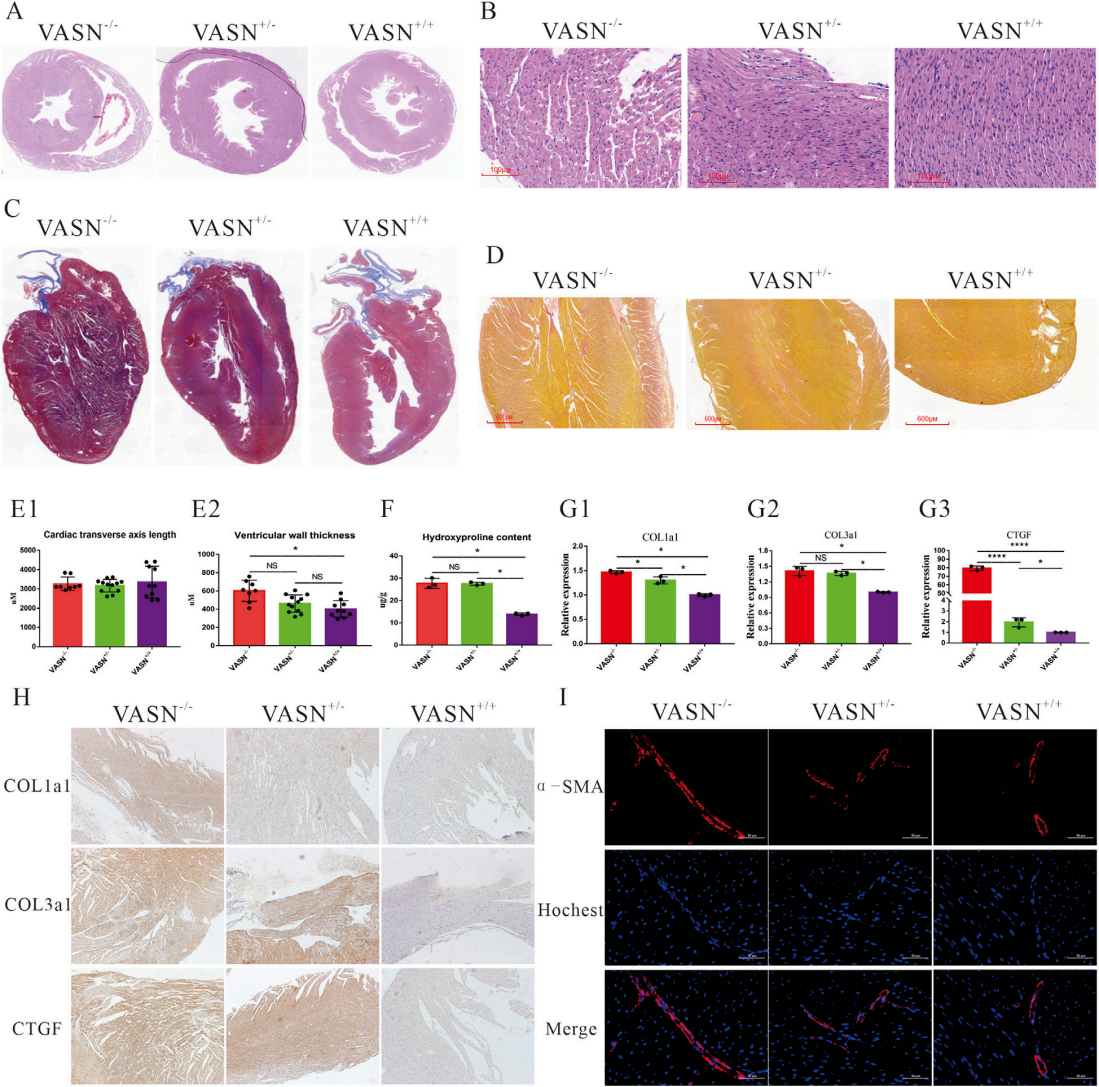
Typical characteristics of myocardial fibrosis (MF) in VASN-deficient mice: **(A)** overall morphology of VASN mouse heart under HE staining; **(B)** changes in the cardiac hypertrophy of VASN mice under HE staining; **(C)** changes in the MF of VASN mice under Masson staining; **(D)** changes in the MF of VASN mice under Sirius staining; **(E1, E2)** changes in the cardiac transverse axis length and ventricular wall thickness in VASN mice; **(F)** changes in the HYP expression levels in VASN mice; **(G1–G3)** changes in the MF markers of VASN mice in qPCR analysis; **(H)** changes in the MF markers of VASN mice in IHC-P analysis; **(I)** changes in the MF markers of VASN mice in IF analysis. *p* < 0.05 indicates significant difference, *p* < 0.0001 indicates extremely significant difference, *p* > 0.05 indicates no difference, and the subtables are represented by superscripts *, ****, and ^NS^.

HE staining showed that the thickness of the heart wall in a VASN^−/−^ mouse was significantly higher than those in VASN^+/+^ and VASN^+/−^ mice (Figures 1A, E2). Significantly higher areas were observed for the cardiac cells of the VASN^−/−^ mice; however, no abnormalities were observed in the heart tissues of the VASN^+/+^ and VASN^+/−^ mice (Figure 1B). These experimental results are consistent with those of our previous report (Sun et al., 2022). Masson and Sirius staining showed that cardiac interstitial fibrosis was significantly enhanced in the VASN^−/−^ mice (Figures 1C, D), but no obvious abnormalities were observed in the heart tissues of the VASN^+/+^ and VASN^+/−^ mice. HYP expressions were significantly increased in the VASN^−/−^ and VASN^+/−^ mice (Figure 1F). qPCR and IHC-P showed that the expression levels of COL1A1, COL3A1, and CTFG were significantly higher in the heart tissues of the VASN^+/+^ and VASN^+/−^ mice (Figures 1G, H). IF analysis showed that the expression level and fluorescence intensity of α-SMA were significantly higher in the VASN-knockout mice (Figure 1I). These results confirmed that the VASN-knockout mice exhibited typical symptoms of MF.

### 3.2 Bioinformatics analysis to explore key molecules involved in MF

**FIGURE 2:**
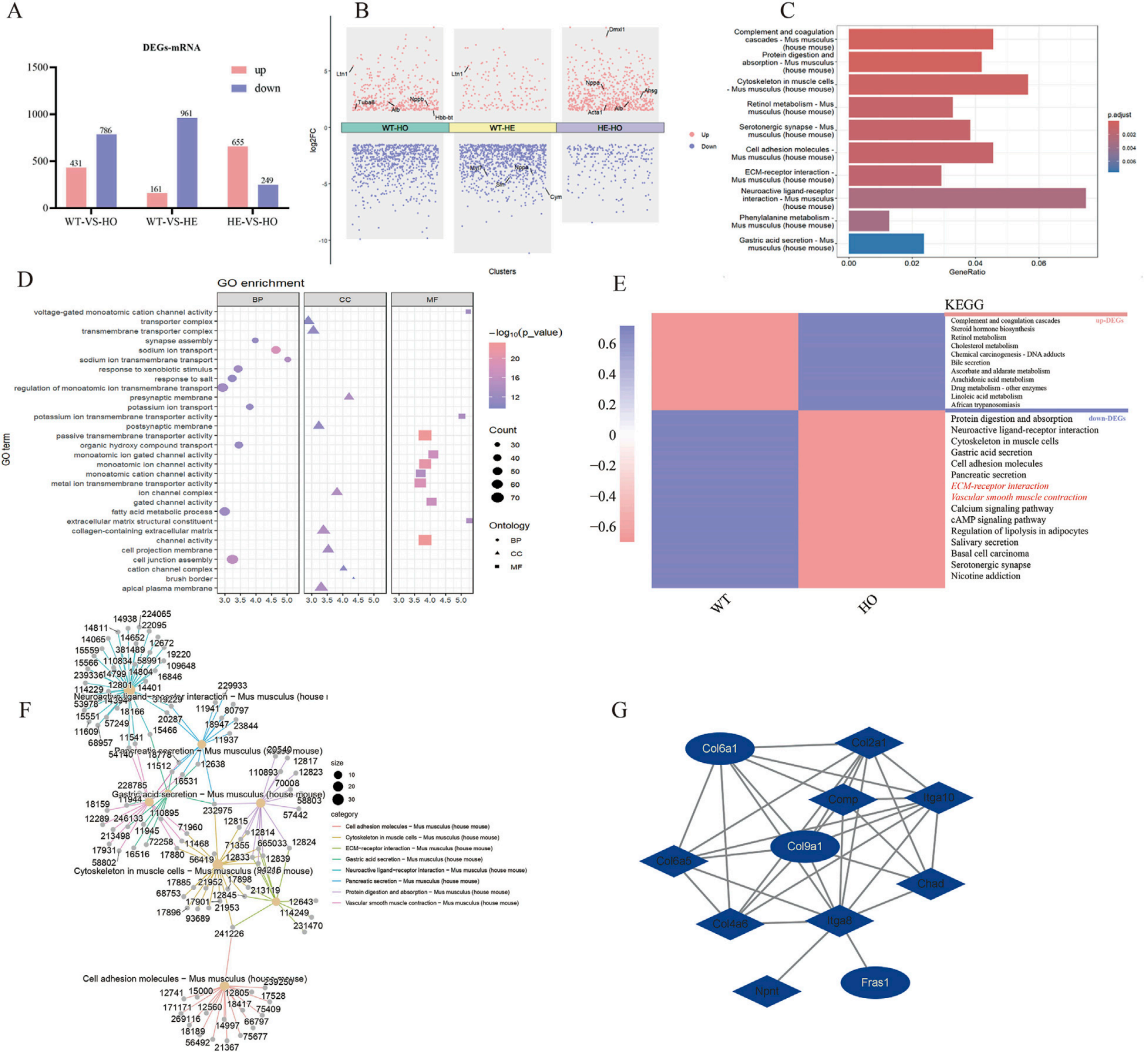
Transcriptome analysis of MF in VASN-deficient mice. The present study entailed heart tissues from VASN^+/+^ (n = 3), VASN^+/−^ (n = 3), and VASN^−/−^ (n = 3) mice for transcriptome sequencing. **(A)** Differentially expressed genes (DEGs) based on miRNA transcriptome data; **(B)** volcano plot presenting the key genes involved in differential expression; **(C)** KEGG enrichment plot of all DEGs in WT-HO; **(D)** GO enrichment map of all DEGs in WT-HO; **(E)** WT-HO differential gene heatmap and KEGG annotation; **(F)** interaction diagram of the top-8 entries in KEGG for all DEGs in WT-HO; **(G)** extracellular matrix pathway gene protein interaction network diagram.

DEGs were identified based on the criteria of a false diagnosis rate (FDR) of <0.05, and |log2 (fold change)| >1.5 (Figures 2A, B). Cluster Profiler (R version 3.5.1, University of Auckland, Auckland, New Zealand) and GO (http://www.geneontology.org; accessed 20 August 2024) were used to enrich and analyze the DEGs. The volcano plot of the DEGs revealed key genes (Figure 2C), among which WT-VS-HO had the highest fold difference and was upregulated. These upregulated genes may be associated with cardiac hypertrophy and fibrosis. The GO enrichment analysis revealed the functional roles of 1,217 DEGs in WT-HO (Figure 2D). Cellular component analysis was used to obtain the localization of the top-10 DEGs. The ECM is one of the main structural components of myocardial tissue, and abnormal expression of the ECM may lead to MF, resulting in cardiac dysfunction.

KEGG enrichment analysis was used to find the top-10 enriched entries for all DEGs, including 786 enriched entries for downregulated genes (Figure 2E); these also recruit genes related to the ECM. According to the interaction diagram of the top-8 downregulated genes in KEGG analysis, *COL6A1* (12,839) and *COL9A1* (12,833) were both involved with the ECM and cytoskeleton in the muscle cell pathways (Figure 2F). Protein–protein interaction (PPI) network mapping of the differential genes in the ECM pathway revealed close interactions between *COL6A1*, *COL9A1*, and *FRAS1* (Figure 2G); here, *COL6A1* and *COL9A1* are upstream genes that regulate *FRAS1* expression via ITGA8.

### 3.3 VASN knockout reduces expression of non-collagen fibers

**FIGURE 3:**
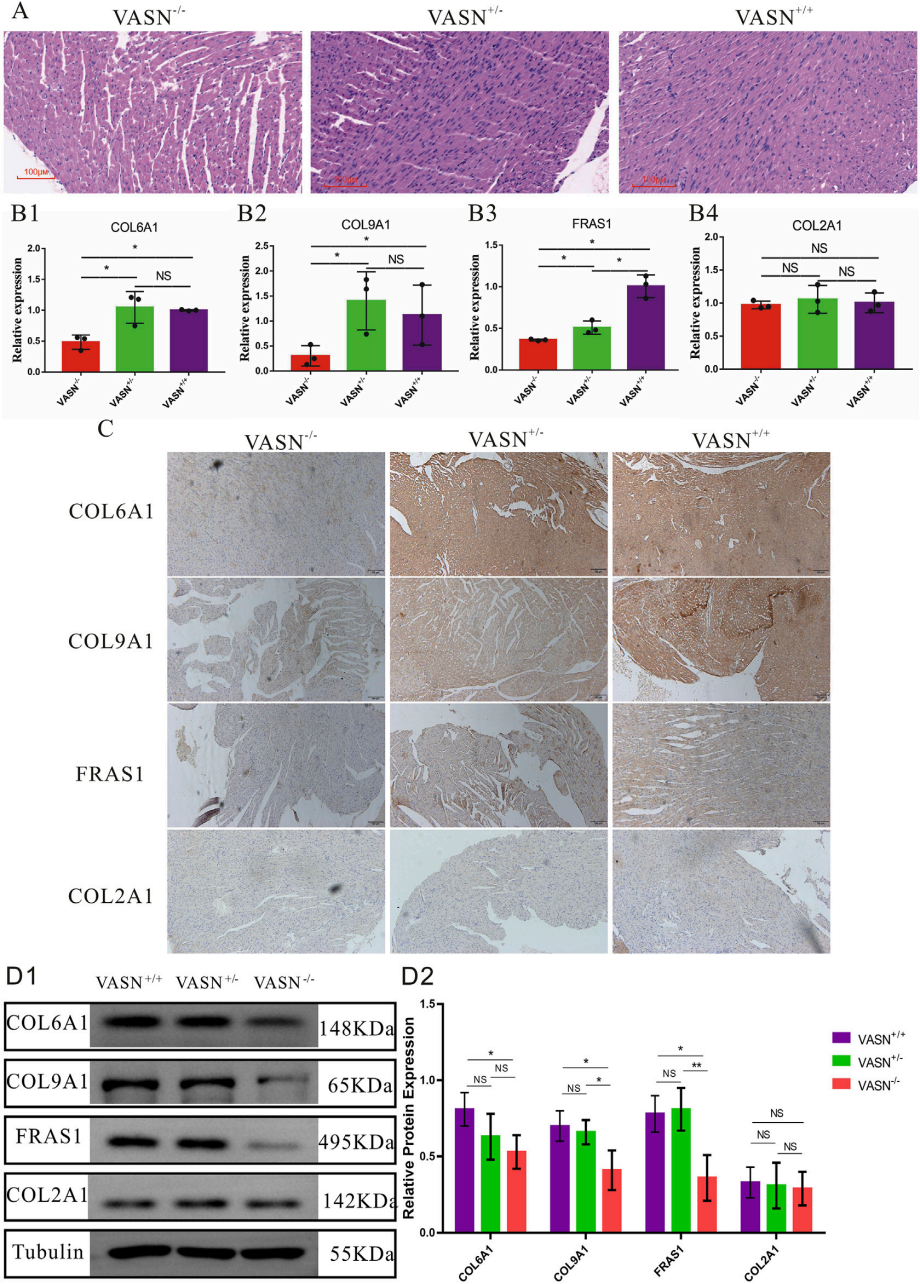
Changes in the non-collagen fibers of MF in VASN-deficient mice. **(A)** Changes in the MF of VASN mice under HE staining; **(B)** qPCR validation of the expression levels of the target genes in heart tissue; **(C)** IHC-P validation of the expression levels of the target genes in heart tissue; **(D)** WB validation of the expression levels of the target genes in heart tissue. *p* < 0.05 indicates significant difference, *p* > 0.05 indicates no difference, and the subtables are represented by superscripts * and ^NS^.

Functional verifications were performed to investigate whether the expression of non-collagen fibers was downregulated in the heart tissue of VASN-knockout mice. HE staining showed that the gaps between the myocardial cells significantly increased in the heart tissue of VASN^−/−^ mice (Figure 3A); qPCR showed that the mRNA expression levels of *CAL6A1*, *CAL9A1,* and *FRAS1* were significantly lower in the VASN^−/−^ hearts (Figure 3B). IHC-P and WB showed that the protein expression levels of CAL6A1, CAL9A1, and FRAS1 were significantly lower in the VASN^−/−^ hearts (Figures 3C, D). These preliminary results imply that the downregulated expression of non-collagen fibers (*CAL6A1*, *CAL9A1,* and *FRAS1*) plays an important role in MF in the VASN^−/−^ mouse hearts.

### 3.4 VASN knockout promotes cardiac inflammation

**FIGURE 4:**
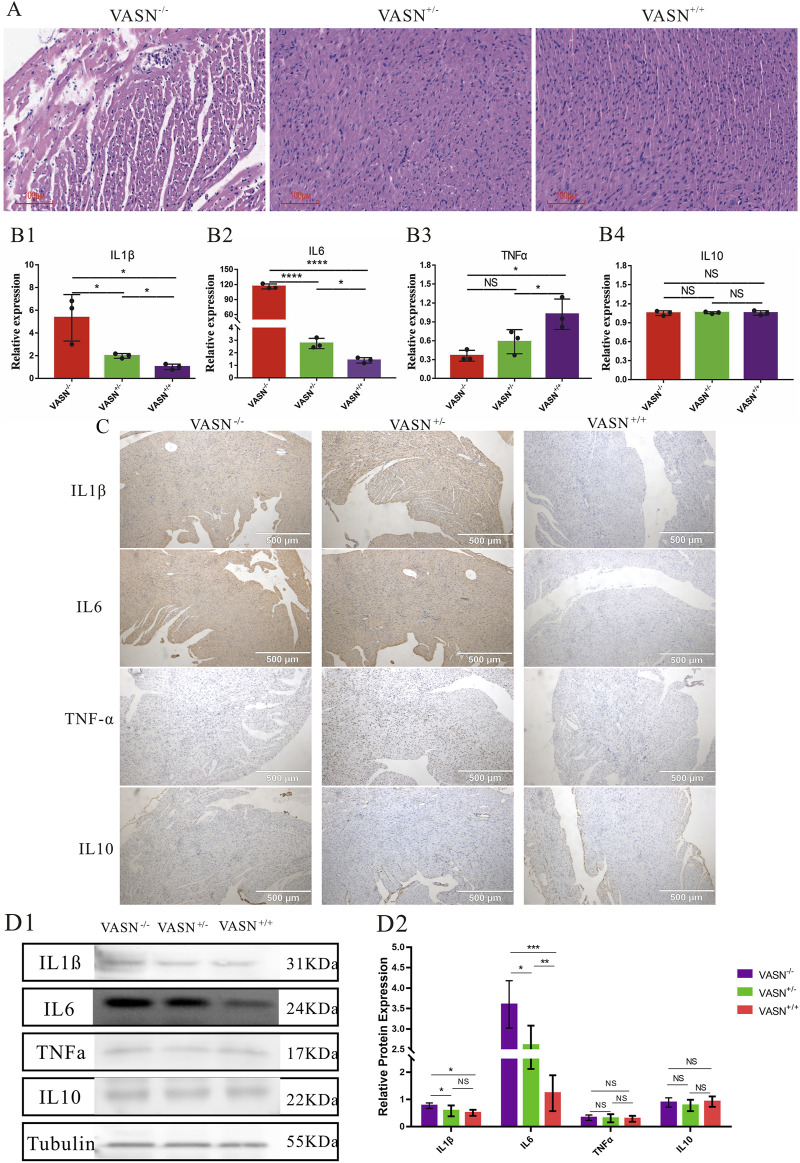
Changes in the inflammatory factors of MF in VASN-deficient mice. **(A)** Changes in the MF of VASN mice under HE staining; **(B)** qPCR validation of the expression levels of the target genes in heart tissue; **(C)** IHC-P validation of the expression levels of the target genes in heart tissue; **(D)** WB validation of the expression levels of the target genes in heart tissue. *p* < 0.05 indicates significant difference, *p* < 0.01 indicates extremely significant difference, *p* < 0.001 indicates extremely significant difference, *p* < 0.001 indicates extremely significant difference, *p* > 0.05 indicates no difference, and the subtables are represented by superscripts *, **, ***, ****, and ^NS^.

To investigate whether myocardial cell inflammation is exacerbated in MF, the inflammatory factors were identified. Accordingly, HE staining showed hypertrophy or atrophy of the myocardial cells, nuclear condensation, diffuse vacuolization of the myocardial cells, myocardial scars, and significantly increased immune cells in the heart tissues of VASN^−/−^ mice (Figure 4A); qPCR showed that the mRNA expression levels of IL1β and IL6 were significantly upregulated in the VASN^−/−^ hearts (Figure 4B). IHC-P and WB showed that the protein expression levels of IL1β and IL6 were significantly higher in the VASN^−/−^ hearts (Figures 4C, D). These results indicate that intensified inflammation could cause MF in VASN^−/−^ mouse hearts.

## 4 Discussion

**FIGURE 5:**
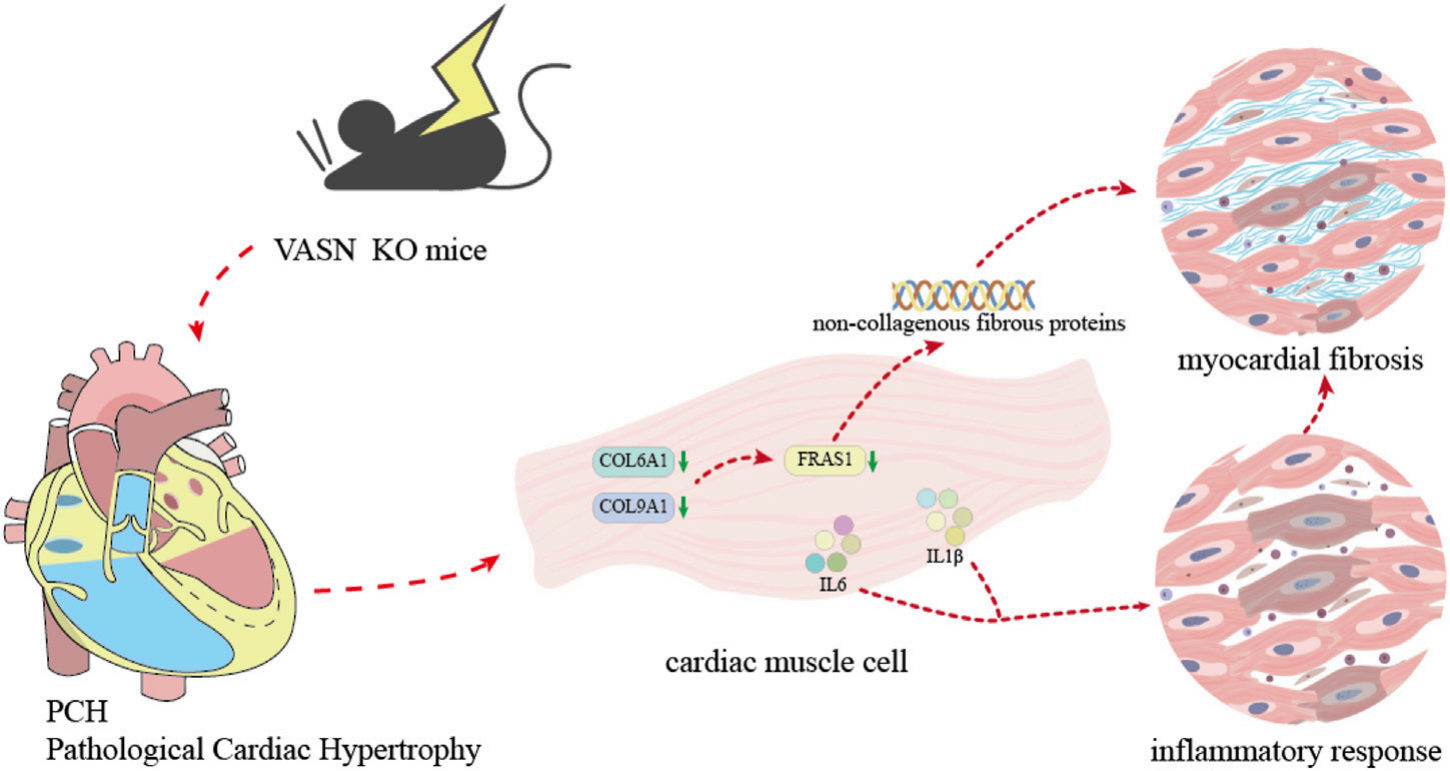
Diagram showing the mechanism by which VASN knockout induces MF by regulating the non-collagen fibers and inflammation.

MF is a complex pathological process that plays a crucial role in the occurrence and development of cardiovascular disease. MF and cardiac hypertrophy often coexist and interact with each other (Pagourelias et al., 2021), and cardiac hypertrophy could lead to MF. Under long-term pressure or increased volume load, the cardiac cells undergo pathological hypertrophy (Figure 5). However, cardiac hypertrophy is often accompanied by remodeling of the myocardial ECM, including collagen deposition and fibrosis (Detterich, 2017). MF caused by cardiac hypertrophy may be closely related to multiple mechanisms, such as activation of the neuroendocrine system (Qiu et al., 2019), inflammatory responses (Bacmeister et al., 2019), and oxidative stress (Wang et al., 2017). MF exacerbates the progression of cardiac hypertrophy as the stiffening and reduced compliance of the fibrotic myocardial tissue impair both diastolic and systolic heart functions (Lafuse et al., 2020). To maintain the pumping function, the cardiac cells are further enlarged, thereby exacerbating the degree of cardiac hypertrophy. MF can also affect the electrophysiological properties of the myocardial cells, thereby increasing the risk of arrhythmias (Baggett et al., 2023).

One of the typical features of MF is the adverse repair response of the cardiac tissue to various damaging factors. HE staining showed that the gaps between the myocardial cells widened during MF and that there was proliferation of pale pink fibrous tissue in the interstitium (Karur et al., 2024). As the degree of fibrosis worsened, the fibrous tissue increased gradually, and focal or diffuse fibrous cord-like structures became more pronounced. Masson staining showed significantly larger blue areas in the MF heart tissue, indicating greater deposition of collagen fibers. The myocardial interstitium in the fibrotic area was stained dark blue, forming a sharp contrast with the red color (muscle fibers) of normal myocardial tissue (Flori et al., 2024). Sirius staining of MF tissue showed large numbers of type I collagen fibers that appeared strongly positive in red or yellow color, whereas type III collagen fibers were relatively fewer and showed lighter staining (Qi et al., 2022). HYP was increasingly expressed in the fibrotic cardiac tissues (Yang et al., 2021). Collagen fiber types I and III are shown to be significantly increased in MF tissues (Xing et al., 2024). The expression level of α-SMA is reported to be low in normal myocardial cells but high in fibrotic cardiac tissues (Hsieh et al., 2022). Our experimental results are consistent with the findings of the above literature, indicating that VASN-knockout mice exhibit typical symptoms of MF.

The *VASN* gene is important for the occurrence and development of cardiovascular diseases. In atherosclerosis, abnormal expression of the *VASN* gene can cause endothelial dysfunction, reduce the resistance of the vascular endothelium to lipid deposition, and promote the formation of atherosclerotic plaques (Louvet et al., 2022). VASN may regulate the expression of adhesion molecules on the surfaces of endothelial cells, increase the adhesion of leukocytes to the vascular walls, and trigger inflammatory reactions to accelerate atherosclerosis (Huang et al., 2015). After myocardial infarction, local tissue ischemia and hypoxia can trigger a series of pathophysiological changes, and VASN is known to be involved in regulating the balance between apoptosis and regeneration of the myocardial cells (Pintus et al., 2018). Abnormal VASN expression leads to increased apoptosis of the myocardial cells, hindered myocardial repair and regeneration capabilities, exacerbated myocardial injury, and pump dysfunction (Shamhart and Meszaros, 2010). Under hypertension, the pressure on the vascular wall increases, and VASN affects the tension and compliance of blood vessels by regulating the contraction and relaxation of the vascular smooth muscle cells (Qin et al., 2024). Owing to dysregulation of VASN expression, the vascular smooth muscles contract excessively, further increasing the blood pressure and exacerbating the burden on the cardiovascular system (Wang et al., 2024).

MF is closely related to the occurrence and development of non-collagenous fibers, which play important roles in normal cardiac tissues. Non-collagen fibers together with collagen fibers form the ECM of the myocardial cells, providing structural support and mechanical stability to the cells (Shamhart and Meszaros, 2010). Non-collagen fibers include various components, such as elastic fibers, fibronectin, and laminin. In MF, changes to the non-collagen fibers often occur before significant changes to the collagen fibers. In the early stages of MF, fibronectin may respond to myocardial injury (Ning et al., 2017). Non-collagen fibers are shown to promote the adhesion, migration, and activation of cardiac fibroblasts, laying the foundation for excessive deposition of collagen fibers (Luther et al., 2012). Elastic fibers endow the myocardium with a certain degree of elasticity in a normal heart, which is beneficial for relaxation and contraction of the heart (Hiesinger et al., 2012). However, these elastic fibers are damaged or replaced by collagen fibers in MF, leading to decreased elasticity and compliance of the heart (Lin et al., 2022). In addition, non-collagen fibers can participate in the regulation of the MF process by interacting with cytokines and growth factors.

Inflammatory factors are another trigger of MF, and there are numerous inflammatory factors that can directly induce MF. Both IL1β and IL6 were observed to play important roles in MF; IL1β activates the nuclear factor kappa B (NF-κB) signaling pathway to promote the production of more profibrotic factors by the cardiac fibroblasts, thereby accelerating MF (Sun et al., 2023); IL6 promotes the activation of cardiac fibroblasts and collagen synthesis through various pathways, such as the downstream signal transduction and transcription activating protein 3 (STAT3) (Rao et al., 2024). MF also triggers inflammatory reactions, resulting in a vicious cycle. As MF progresses, the structure and functions of the myocardial tissue induce local tissue hypoxia, metabolic disorders, and other conditions. Inflammatory cells such as macrophages then aggregate in the fibrotic myocardial tissues, releasing more inflammatory factors like IL1β and IL6 to exacerbate the severity of MF (Fu et al., 2024). The inflammatory factors interact with other signaling pathways to promote MF; they also affect the survival and functions of the myocardial cells, thereby promoting the development of MF (Liu et al., 2020).

VASN deletion leads to MF in mice with cardiac hypertrophy. VASN-knockout mice exhibit typical pathological, imaging, and molecular features of MF. Differential analysis of the various genes involved, especially the ECM genes (*COL6A1*, *COL9A1,* and *FRAS1*), showed strong correlations even as their expression levels decreased significantly in the heart tissues of VASN-knockout mice. The expression levels of inflammatory factors IL1β and IL6 were significantly upregulated in the heart tissues of VASN-knockout mice. These preliminary results reveal that VASN knockout leads to MF by downregulating the non-collagen fibers and promoting inflammation.

## Data Availability

The data presented in the study are deposited in the Harvard Dataverse repository, Harvard Dataverse tracking link https://doi.org/10.7910/DVN/NDTQEP. Further inquiries can be directed to the corresponding author.
